# Duodenal-Distal Ileal Fistula After Laparoscopic Radical Right Hemicolectomy: A Case Report

**DOI:** 10.3389/fsurg.2022.851348

**Published:** 2022-03-03

**Authors:** Xiaolan You, Xiaojun Zhao, Chuanjiang Huang, Zhiyi Cheng, Guiyuan Liu, Xianhe Shen, Tingrui Zheng

**Affiliations:** ^1^Taizhou Clinical Medical School of Nanjing Medical University (Taizhou People's Hospital), Taizhou, China; ^2^Dalian Medical University, Dalian, China

**Keywords:** right hemicolectomy, duodenal-distal ileum fistula, short bowel syndrome, diarrhea, weight loss, case report

## Abstract

Right hemicolectomy for colon cancer may be complicated by leaks, stenoses, or fistulas. These complications usually occur at the ileocolic anastomosis and can be managed endoscopically. However, fistulas that are large cannot be managed by endoscopy and require surgical intervention. After laparoscopic radical right hemicolectomy, duodenal fistulae is relatively rare. Among duodenal fistulae, internal duodenocolic fistulae is relatively common, but duodeno-ileum fistulae is extremely rare. Here, we report a case of duodeno-distal ileum fistula after right hemicolectomy with short bowel syndrome, that was surgically treated. After surgical treatment, the symptoms of short bowel syndrome disappeared, weight gain was obvious, and the clinical effect was satisfactory.

## Case Report

**Figure 1:**
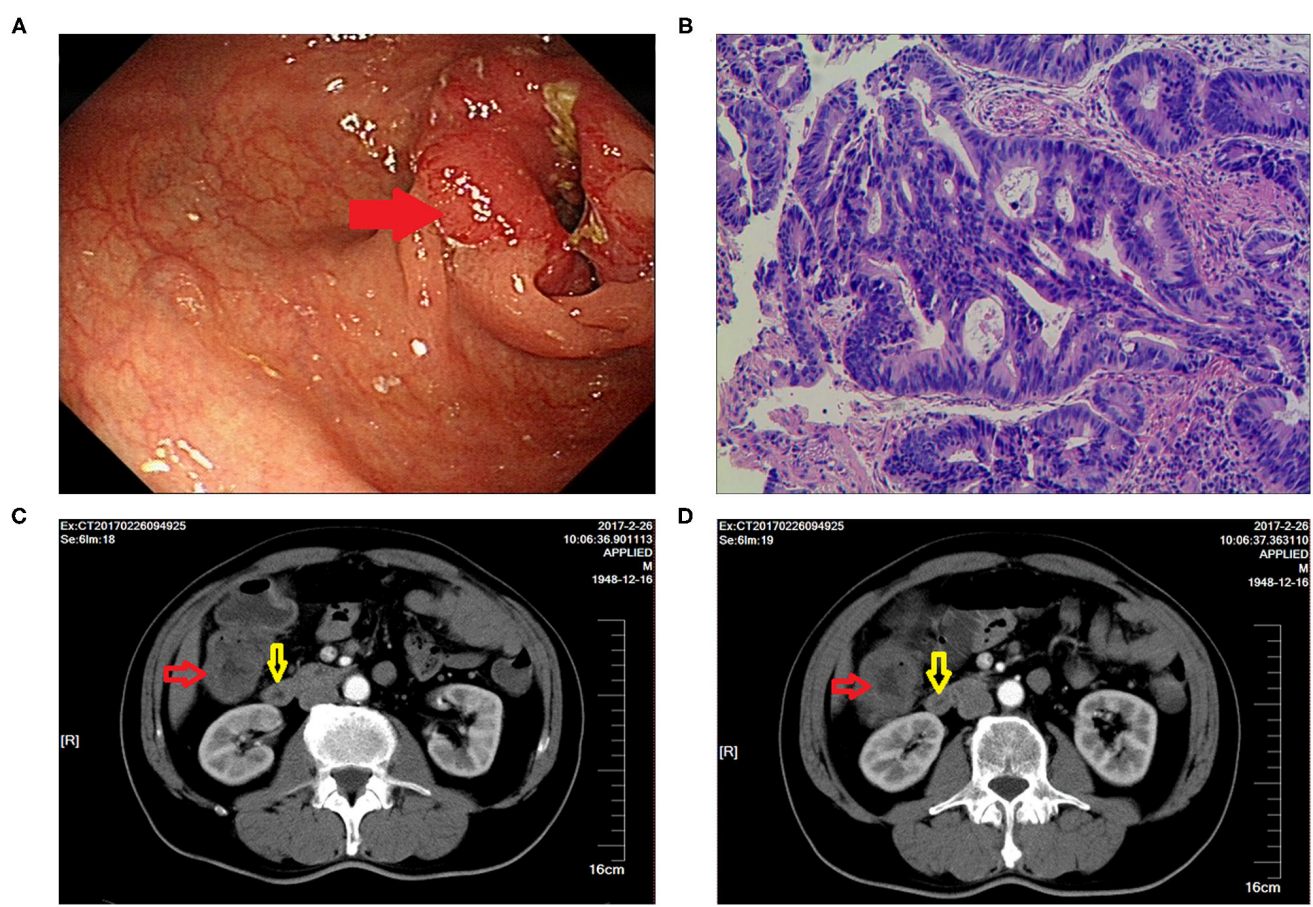
Imaging findings for right hemicolectomy performed 4 years ago. **(A)** Colonoscopy showed the presence of a colonic tumor (indicated by the red arrow). **(B)** H&E staining was indicative of adenocarcinoma. **(C,D)** CT showed a tumor of hepatic flexure of colon that did not invade the duodenum (the red arrow indicates the tumor, and the yellow arrow indicates the duodenum).

**Figure 2:**
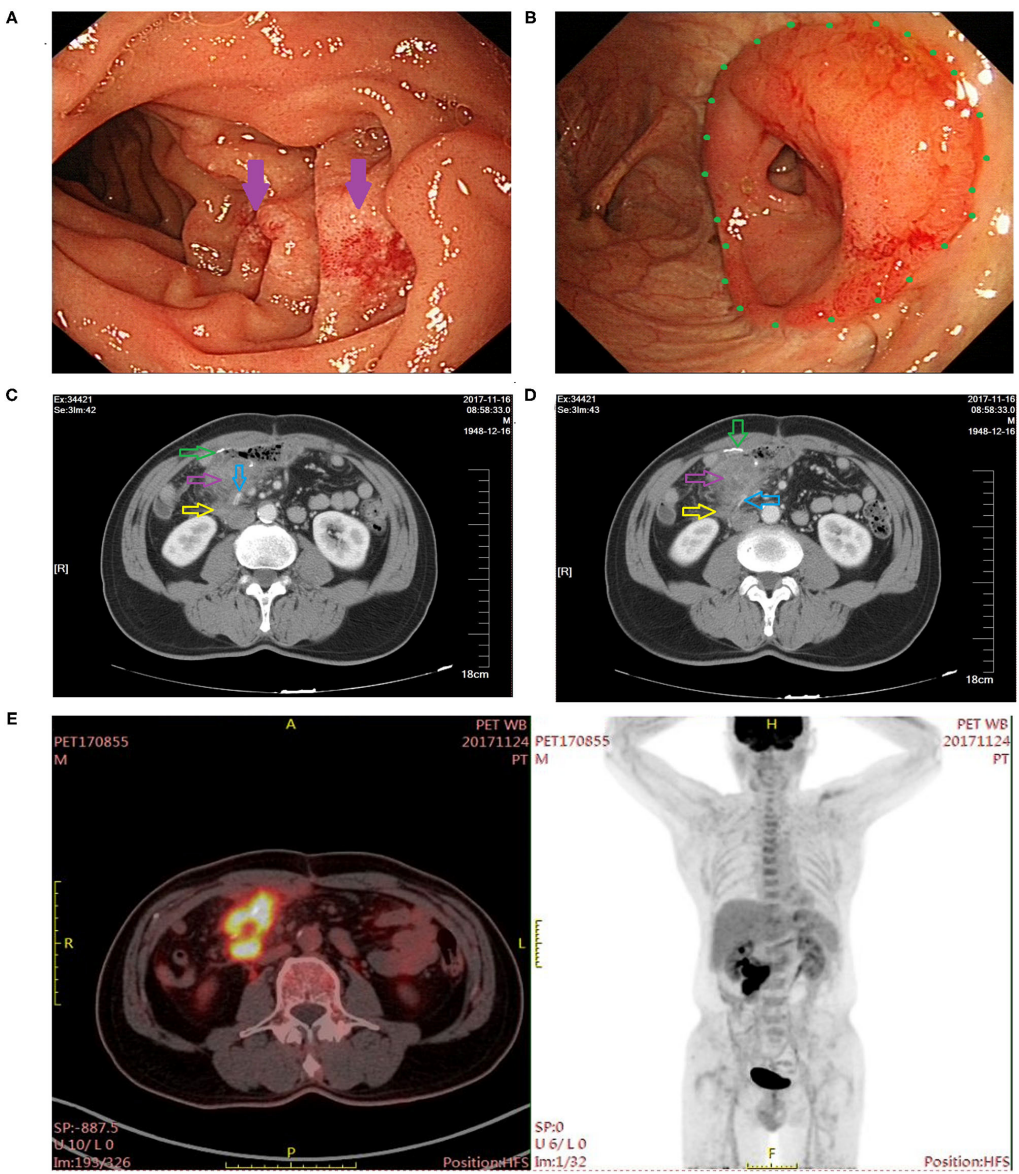
Postoperative imaging findings at 8 months after right hemicolectomy. **(A)** Gastroscopy revealed inflammatory changes in the descending part of the duodenum (indicated by the purple arrow). **(B)** Colonoscopy showed inflammatory changes at the ileocolic anastomosis site (indicated by the green dot). **(C,D)** CT examination showed exudation around the ileocolic anastomosis (the green arrow indicates the ileocolic anastomosis; the purple arrow indicates inflammatory exudate; the yellow arrow indicates the duodenum; and the blue arrow indicates the vascular clip). **(E)** PET-CT showed high concentration of radioactivity around the anastomosis.

A 72-year-old male patient presented with frequent diarrhea after eating and lost more than 5 kg of body weight in the past 3 month, who underwent radical resection for right colon cancer more than 4 years ago. In February 2017, the patient was diagnosed with colon tumor by colonoscopy (Figure 1A), and pathological examination of the biopsy specimen indicated adenocarcinoma (Figure 1B). Computer tomography (CT) showed a tumor of hepatic flexure of colon, and the tumor did not invade the duodenum (Figures 1C,D). In March 2017, the patient underwent laparoscopic radical right hemicolectomy at another hospital. Postoperative pathological examination revealed a poorly differentiated colonic adenocarcinoma and stage T4AN1M0 (stage IIIA). One month after surgery, the patient received four courses chemotherapy, regimen comprising 150 mg oxaliplatin (ivgtt, d1) and 1,500 mg capecitabine (oral, BID, 1–14 day), one course of treatment is 21 days. In November 2017, the patient underwent gastroscopy, and inflammatory change was observed in the descending part of the duodenum (Figure 2A). Colonoscopy revealed inflammatory change in the anastomosis (Figure 2B), CT showed edema and exudation around the ileocolic anastomosis, and the postoperative CT scan showed a vascular clip in preduodenal space and that was adjacent to the duodenum wall (Figures 2C,D). Additionally, positron emission tomography (PET)-CT revealed a high concentration of radioactivity around the anastomosis (Figure 2E). Since there were no signs of cancer recurrence or metastasis, the patient did not undergo regular examination and follow-up from then on. In April 2021, the patient developed diarrhea after eating (more than 10 stools per day), accompanied by rapid weight loss of more than 5 kg in 3 months.

The patient had no history of specific chronic or infectious diseases, and had no familial diseases associated with the present illness. Physical examination: On admission, the patient showed signs of malnutrition, and his body mass index (BMI) was 20.07, physical examination and laboratory tests did not reveal any other observations of concern.

**Figure 3:**
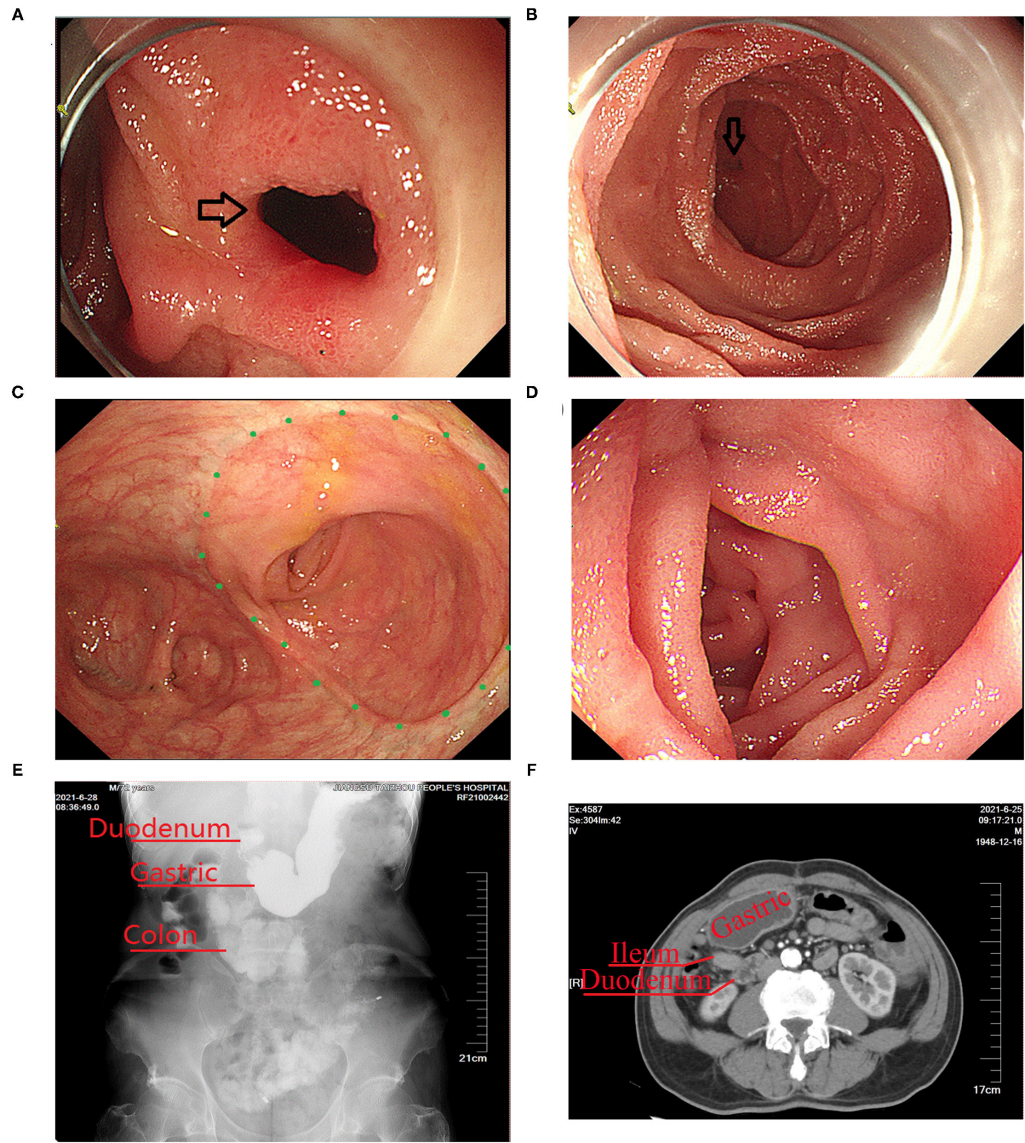
Imaging findings obtained before surgical treatment of the duodeno-distal ileal fistula. **(A,B)** Gastroscopy showed a fistula in the descending part of the duodenum (the black arrow indicates the fistula). **(C)** Colonoscopy showed that there were no abnormalities in the ileocolic anastomosis (indicated by the green dot). **(D)** Colonoscopy showed that there were no abnormalities in the terminal ileum. **(E)** Upper gastrointestinal imaging examination showed that after passing through the duodenum, the contrast agent directly entered the lower colon. **(F)** CT scan showed that there was no obvious mass around the anastomosis and no inflammatory exudation.

Gastroscopy revealed a fistula in the descending part of the duodenum with a diameter of 8 mm, but no tumor was observed around the fistula (Figures 3A,B). Colonoscopy showed that there were no abnormalities in the residual colon and anastomotic site (Figure 3C), and no obvious fistula was observed in the terminal ileum 10 cm from the ileocolic anastomotic site (Figure 3D). Upper gastroenterography showed that after passing through the duodenum, the contrast agent directly entered the colon (Figure 3E). CT showed that there was no obvious mass around the anastomosis and no inflammatory exudate (Figure 3F).

Comprehensive analysis indicated that the patient had a duodenal-distal ileum fistula and short bowel syndrome that probably developed after laparoscopic radical right hemicolectomy.

**Figure 4:**
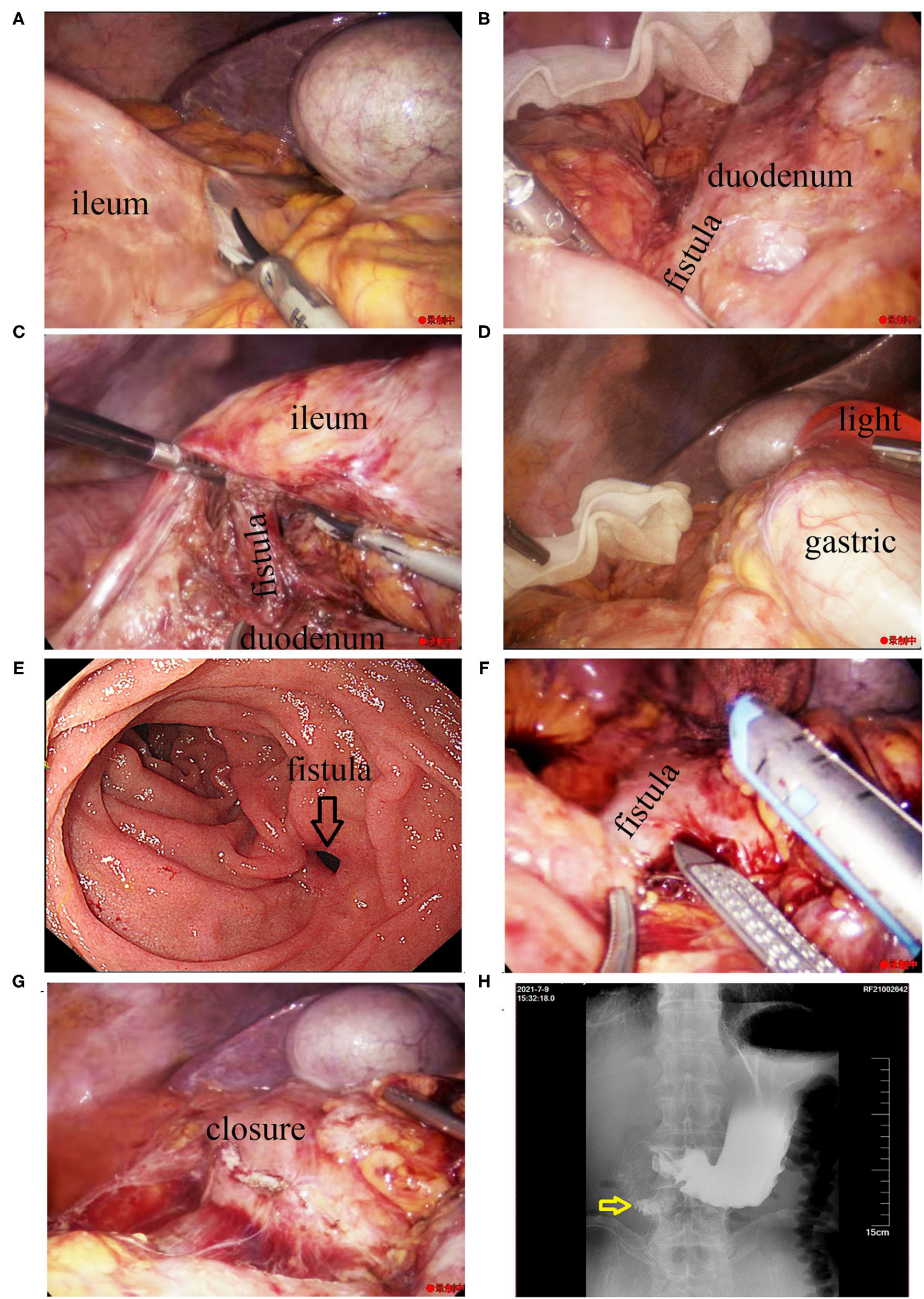
Intraoperative imaging findings during laparoscopic treatment of the fistula. **(A)** The small intestine was observed to have adhered to the right upper abdomen. **(B)** A tubular structure was seen in the lateral part of the lower duodenum, connecting to the distal ileum. **(C)** The tubular structure can be seen from the rear of the mesentery. **(D)** Insertion of the gastroscope into the duodenal bulb. **(E)** Gastroscopic image showing the fistula. **(F)** The fistula was cut and closed with a linear cutting stapler. **(G)** The closure was strengthened with a barb suture. **(H)** Postoperative imaging findings after surgical treatment of the fistula. Upper gastrointestinal imaging examination showed there was no leakage of the contrast agent in the duodenum 7 days after the laparoscopic procedure (the yellow arrow indicates the location of the original duodenal fistula).

Since the large diameter of the fistula, endoscopic treatment was not feasible. Instead, surgical treatment was administered after discussion with a multi-disciplinary team. After preoperative examination to exclude any surgical contraindications, laparoscopic exploration was performed under general anesthesia On July 2, 2021. During the procedure, a part of the small intestine was adhered to the right upper abdomen (Figure 4A). The fistula was observed as a tubular structure in the lateral part of the descending duodenum, that connected to the distal ileum (Figure 4B). After the small intestine was separated, the duodenal bulb and descending part of the duodenum in the preduodenal space were freed. Enter the original Tolt's space from the rear of the mesentery, then dissociated the adhesion of the horizontal part of duodenum. Finally, only a tubular structure was found connecting the anterior lateral wall of the descending duodenum and the ileum 15 cm away from the anastomotic site (Figure 4C). Intraoperative gastroscopy confirmed that the tubular structure was the duodenal fistula (Figures 4D,E). A linear cutting stapler (Ezisurg Medical Co. Ltd., China) was used to cut and close the fistula, and a barb suture was used to strengthen the closure (Figures 4F,G).

During the surgery, the gastric tube was placed in the duodenum to ensure effective gastrointestinal decompression. The patient was required to fast, and parenteral nutritional support was provided after the procedure. Postoperative day 7, upper gastroenterography confirmed no fistula in the duodenum (Figure 4H). The patient was placed on a fluid diet, then semi-fluid diet from the next day, and discharged successfully on the postoperative day 11. At the 6-month follow-up after discharge, the patient was on a normal diet, had no abdominal pain or diarrhea, and had gained about 5 kg of body weight.

## Discussion

Enteroenteric fistulae often secondary to benign or malignant disease, such as colon cancer (1), Crohn's disease (2), radiation enteritis (3), peptic ulcer disease (4, 5), and acute bowel ischemia (6), but duodenal fistulae are relatively rare. Additionally, among duodenal fistulae, internal duodenocolic fistulaes are relatively common, but duodeno-ileum fistulaes are rare. Enteroenteric fistulae are generally non-iatrogenic, and iatrogenic enteroenteric fistulaes are rare (7). Here, we report a rare case of a duodenal-distal ileum fistula of iatrogenic origin that developed 4 years after laparoscopic radical right hemicolectomy.

In the present report, we describe a case of benign duodenal fistula with no evidence of cancer recurrence or metastasis in a patient who had undergone laparoscopic radical right hemicolectomy 4 years ago. Benign duodenal fistulae usually occur secondary to duodenal ulcer (8), Crohn's disease (9), cholelithiasis (10), and tuberculosis (11), and is rarely secondary to iatrogenic injury. However, the patient in this case did not have a history of duodenal ulcer, Crohn's disease, or any other chronic disease. As he did have a history of other surgery, so he was diagnosed with iatrogenic benign duodenal fistula.

Duodenal fistulas with long intestinal tubes were putted-aside may result in short bowel syndrome symptoms, such as diarrhea, metabolic, nutritional disorders, and weight loss as reported in the present case. The diarrhea in such cases is associated not only with mechanical defects, but also with colonic bacterial contamination of the upperdigestive. This is because colon contents passes through the fistula and into the duodenum, and this reflux leads to bacterial overgrowth and infectious diarrhea (12). Subsequently, frequent diarrhea results in malnutrition and dramatic weight loss. Epigastric pain and vomiting are other important symptoms of duodenal fistula. Epigastric pain is usually cause by a primary disease and metabolic syndrome is caused by the internal fistula, epigastric pain may be accompanied by feculent vomit or fecal gastric contents due to reflux (12). The patient in this case had no symptoms of epigastric pain or vomiting. The absence of vomiting may be related to the location of the fistula, as it was present the descending part of the duodenum, and the pylorus prevents stool from entering the stomach by reflux. Alternatively, as the other end of the fistula was present in the distal ileum rather than the colon, the ileum stores less feces, fecal reflux into the duodenum was probably not easy.

In patients with intestinal fistula with short bowel syndrome and nutritional disorders, the diagnosis must be based on the clinical manifestations of the patient, combined with the findings of endoscopic and radiological examination (13). In this case, gastroscopy revealed a leak in the descending part of the duodenum, but colonoscopy showed that there were no obvious fistulas in the residual colon and terminal ileum 10 cm from the ileocolic anastomosis. However, upper gastroenterography showed that the contrast agent entered the colon immediately after entering the duodenum. Based on the patient's diarrhea symptoms and the results of colonoscopy, the clinical diagnosis of duodenal-ileal fistula was considered. Intraoperative anatomical confirmed that the fistula was located between the lateral part of the descending duodenum and the ileum about 15 cm from the anastomosis. Therefore, the preoperative diagnosis was correct.

Secondary duodenal fistula after right semicolon surgery is rarely reported, especially duodenal-ileal fistulae, which has not been reported so far. In this case, no tumor-like tissue was observed around the fistula intraoperatively. Therefore, local recurrence of the tumor was ruled out as a cause of the fistula. The influence of postoperative adjuvant chemotherapy on the anastomotic leakage has not been reported. However, previous studies have shown that preoperative neoadjuvant chemotherapy did not increase the incidence of anastomotic leakage of the colorectal cancer (14), so we considered the occurrence of postoperative delayed fistula in this patient was not related to postoperative adjuvant chemotherapy. Gastroscopy examination of the patient 8 months after surgery showed descending duodenal erosion, CT and PET-CT showed inflammatory changes around the terminal ileum. Therefore, it was considered that the duodenal-ileum fistula was caused by a local chronic infection. A postoperative CT scan of the patient showed a vascular clip adjacent to the fistula of duodenum. There was no damage to the intestinal wall behind the vascular clip, and although possible, there is no evidence to indicate a causal relationship between the occurrence of duodenal-ileal fistula and the compression of the vascular clip. As the patient exhibited symptoms 4 years after the primary surgery, the chronic inflammation may have gradually eroded the fistula and mainly occurred between the intestinal walls. No serious edema or obstruction of the intestinal mucosa was observed, so the patient had no obvious abdominal pain or other uncomfortable symptoms.

The method of treatment is dependent on the nature of the fistula (benign or malignant), the location of the fistula, the size of the fistula, and the patient's clinical symptoms (13). The most commonly used treatment methods include endoscopic therapy and surgical treatment. Endoscopic therapy includes endoscopic clipping and endoscopic stent placement, as well as innovative endoscopic techniques, such as suturing (15), vacuum therapy (16), and endoscopic internal drainage (17). However, surgical treatment is the only option for endoscopically challenging fistulas, although surgical interventions may be difficult and associated with a high risk of morbidity and mortality (18). The diameter of the duodenal fistula in the present patient was about 8 mm, and the surrounding mucosal tissue of the fistula was stiff. This made endoscopic clipping or suturing difficult. Therefore, laparoscopy was used to cut off the fistula, and the incision was sutured.

## Conclusion

Duodenal-ileal fistula can be secondary to local chronic infection after radical right hemicolectomy. Large fistulas cause symptoms such as diarrhea and wasting, and the symptoms of malnutrition can worsen if left untreated. In the case of large fistulae that cannot be treated endoscopically and that are not surrounded by inflammatory edema, it is safe, effective, and feasible to cut off and close the fistula to restore intestinal function.

## Data Availability Statement

The original contributions presented in the study are included in the article/supplementary material, further inquiries can be directed to the corresponding author.

## Author Contributions

XY, XZ, XS, and TZ collected the patient's clinical data and wrote the manuscript. XY, XZ, CH, ZC, and GL helped in the management of the patient and collected the patient's clinical data. XY reviewed the literature and wrote the manuscript. All authors read and approved the final manuscript.

## Conflict of Interest

The authors declare that the research was conducted in the absence of any commercial or financial relationships that could be construed as a potential conflict of interest.

## Publisher's Note

All claims expressed in this article are solely those of the authors and do not necessarily represent those of their affiliated organizations, or those of the publisher, the editors and the reviewers. Any product that may be evaluated in this article, or claim that may be made by its manufacturer, is not guaranteed or endorsed by the publisher.
